# Gastric adenocarcinoma arising from hamartomatous inverted polyp during 8‐year follow‐up

**DOI:** 10.1002/deo2.16

**Published:** 2021-08-22

**Authors:** Takuma Okamura, Yugo Iwaya, Tadanobu Nagaya, Futoshi Muranaka, Hiroyoshi Ota, Takeji Umemura

**Affiliations:** ^1^ Department of Gastroenterology Shinshu University School of Medicine Nagano Japan; ^2^ Department of Surgery Shinshu University School of Medicine Nagano Japan; ^3^ Department of Biomedical Laboratory Sciences School of Health Sciences Shinshu University School of Medicine Nagano Japan; ^4^ Department of Life Innovation Institute for Biomedical Sciences Shinshu University Nagano Japan

**Keywords:** gastric cancer, hamartomatous inverted polyp, heterotopic gastric mucosa, long‐term, submucosal tumor

## Abstract

Gastric hamartomatous inverted polyp (GHIP) is rare, with few reports of carcinogenesis from GHIP during long‐term follow‐up. A 51‐year‐old woman was diagnosed as having a submucosal tumor (SMT) during esophagogastroduodenoscopy (EGD) in 2008. In 2016, although the size and height of the lesion had not changed, she was referred to our hospital for further investigation of the lesion. EGD depicted a gastric SMT of 20 mm in diameter in the greater curvature of the upper gastric body, and a biopsy specimen showed a well to poorly differentiated adenocarcinoma. Following successful laparoscopic total gastrectomy, histopathological examination revealed an intramucosal adenocarcinoma arising in GHIP.

## INTRODUCTION

Gastric hamartomatous inverted polyp (GHIP) is characterized pathologically by a submucosal mass of lobulated gastric mucosa, which has a centrally located lumen and is surrounded by smooth muscle communicating with the muscularis mucosa.^1^ Although associations between GHIP and gastric cancer have been reported,^2^ few include observation over a long‐term period. We herein describe the rare case of gastric adenocarcinoma arising from GHIP during 8‐year follow‐up.

## CASE REPORT

**FIGURE 1:**
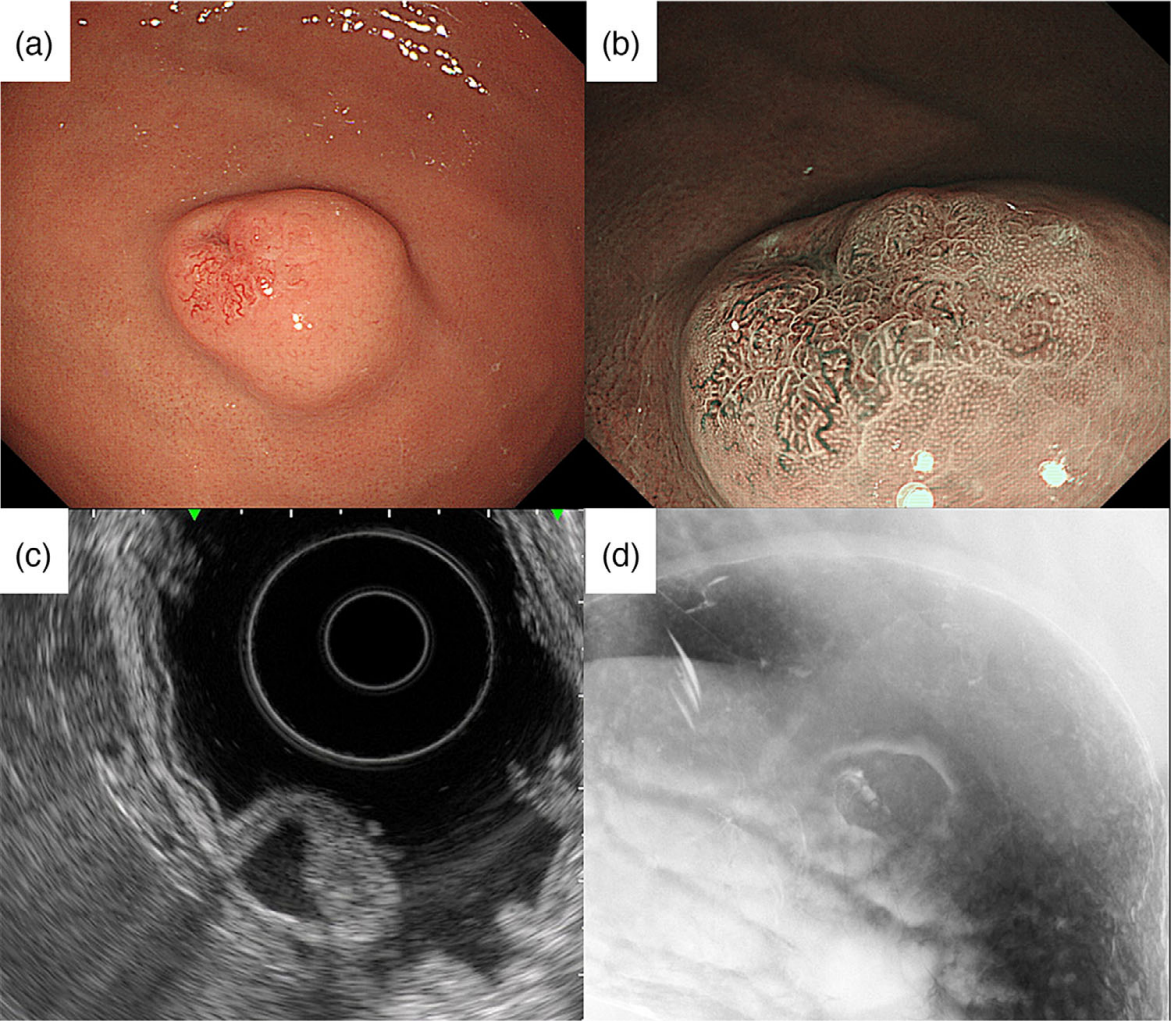
(a) EGD revealed a gastric SMT of 20 mm in diameter in the greater curvature of the upper gastric body with a depression and dilated vessels. (b) Magnifying endoscopy with narrow‐band imaging detected a redness area around the depression having a heterogeneous arrangement of irregular villi and pit pattern without a clear demarcation line. (c) Endoscopic ultrasonography disclosed a heterogeneous tumor with cystic areas located in the third layer. (d) Upper gastrointestinal X‐ray series showed a SMT in the upper gastric body with an irregular depression on its surface

**FIGURE 2:**
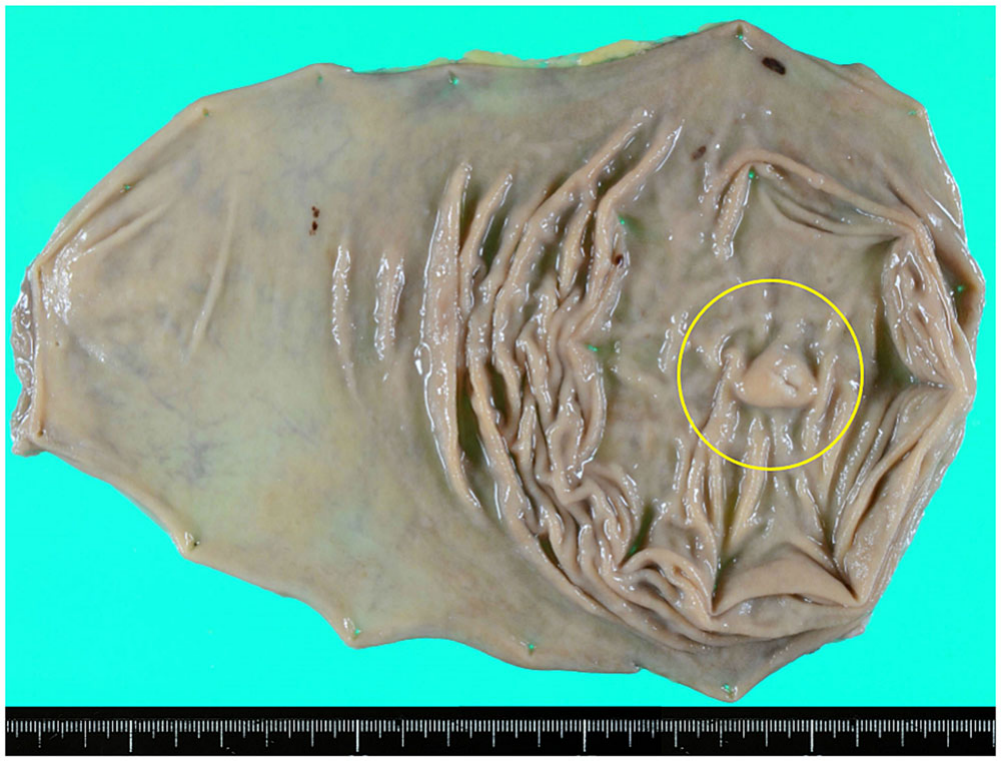
Surgical specimen revealed an 18 × 13 mm SMT with a depression at the top (circle)

**FIGURE 3:**
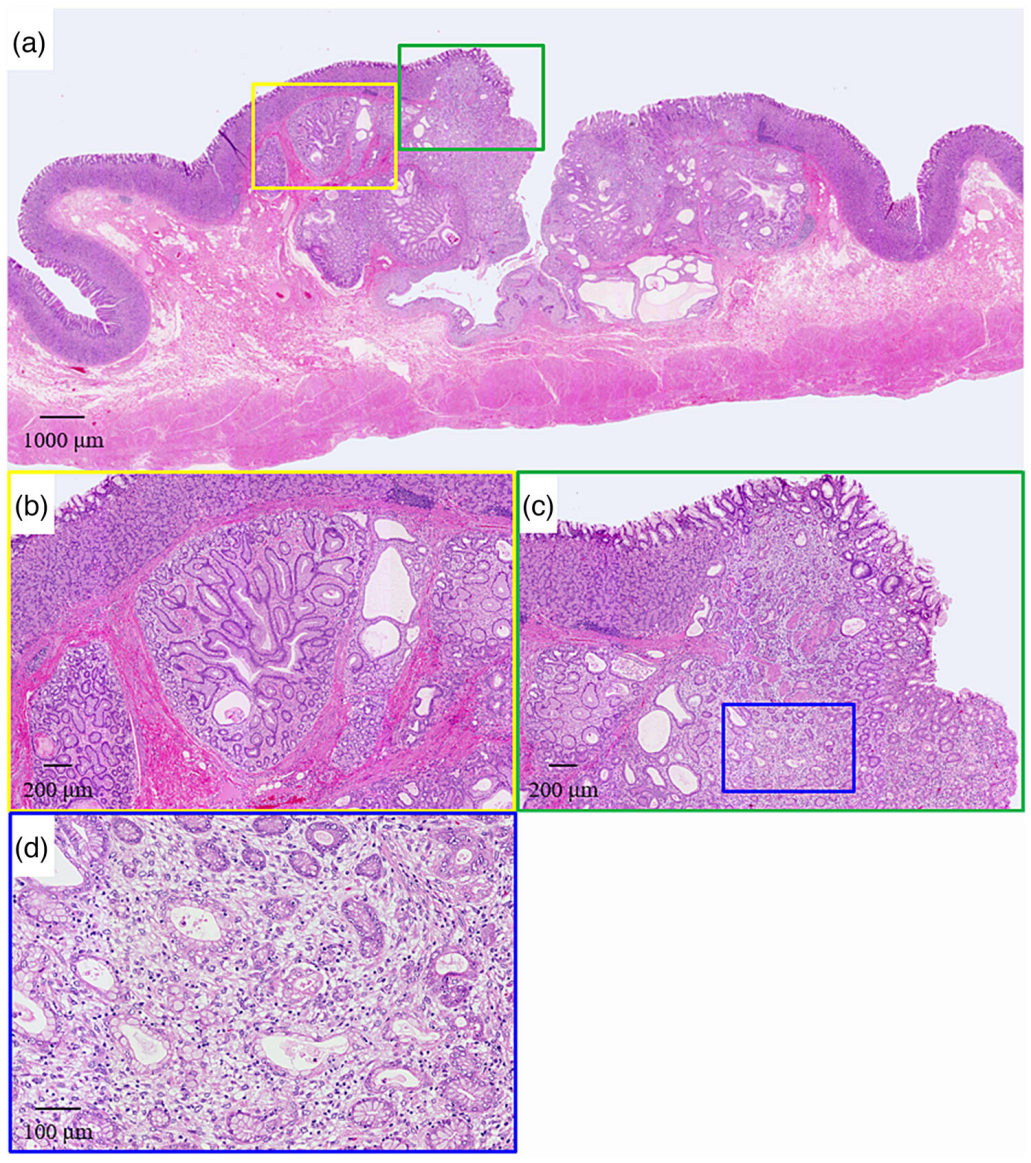
(a) Low magnification (20× magnification) of the SMT showed a submucosal mass of lobulated mucosa of gastric pyloric type, which has a centrally located lumen and is surrounded by smooth muscle communicating with the muscularis mucosa. (b) Magnified image (40× magnification) of the yellow square that was diagnosed as GHIP. (c) Magnified image (40× magnification) of the green square. A well to poorly differentiated adenocarcinoma and signet ring cell carcinoma were observed. (d) Magnified image (100× magnification) of the blue square. A poorly differentiated adenocarcinoma and signet ring cell carcinoma were clearly evident (hematoxylin and eosin staining)

**FIGURE 4:**
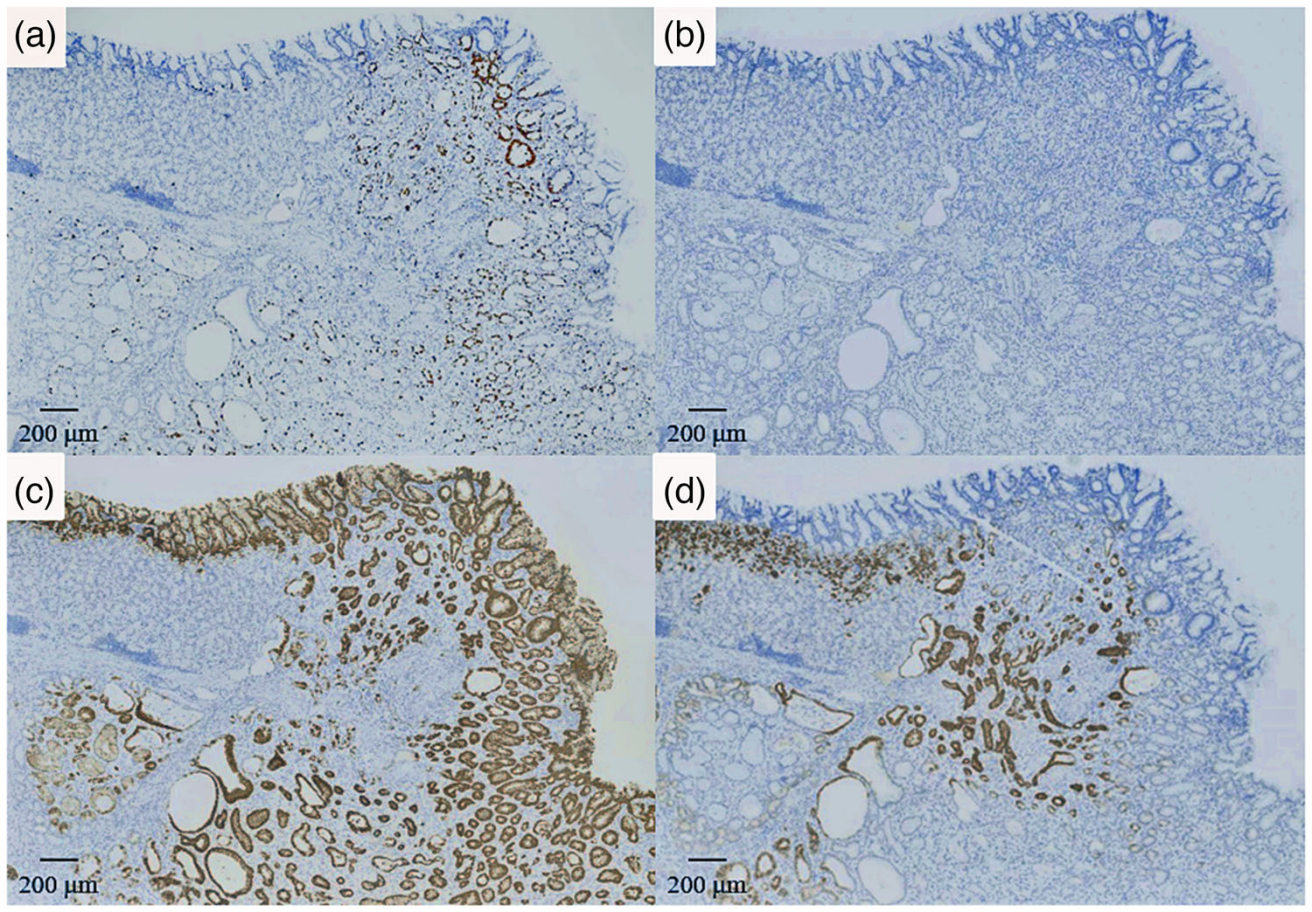
Immunohistochemistry findings (40× magnification). (a) Ki67 staining. (b) MUC2 staining. (c) MUC5AC staining. (d) MUC6 staining. Increased expression of Ki67 was observed in the carcinoma. Both the GHIP and gastric cancer were positive for MUC5AC and MUC6 but negative for MUC2

**FIGURE 5:**
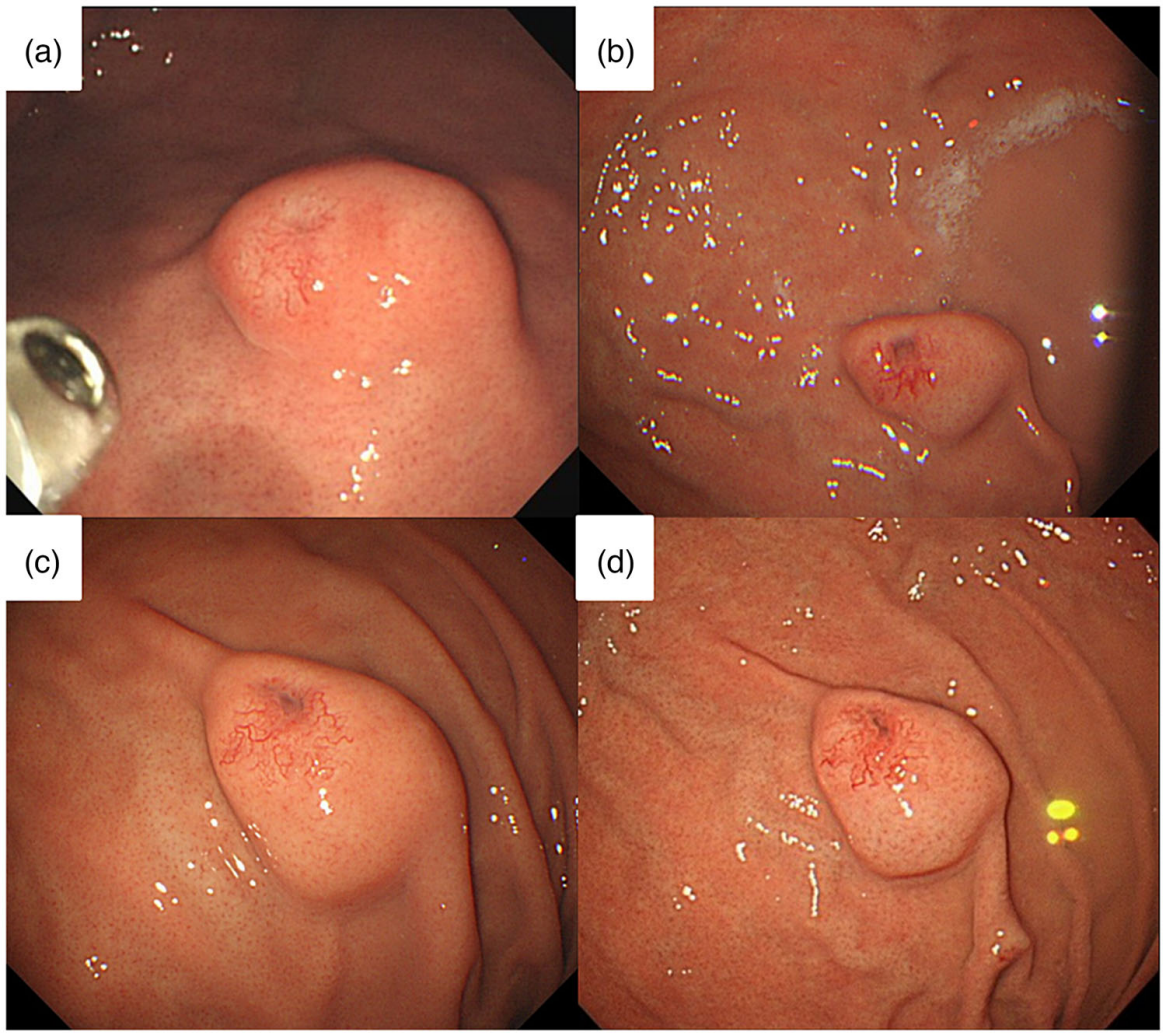
Serial EGD examination results prior to diagnosis: (a) 8 years prior, (b) 5 years prior, (c) 3 years prior, and (d) 1 year prior. The size and height of the lesion did not change remarkably

A 51‐year‐old woman was referred to our hospital in 2016 for further examination of a submucosal tumor (SMT) on esophagogastroduodenoscopy (EGD) first detected in 2008. The patient had undergone annual EGD to follow‐up with no remarkable findings. A biopsy specimen taken in 2011 also showed no evidence of malignancy. On presentation, she had no clinical symptoms, family history, or laboratory data abnormalities, including CEA and CA19‐9. Serum *Helicobacter pylori* antibody was negative. EGD identified a gastric SMT of 20 mm in diameter with a positive cushion sign in the greater curvature of the upper gastric body. The lesion exhibited a depression suspected as the crypt opening and dilated vessels with a branching architecture around the depression on the proximal side of the lesion (Figure 1a). There were no atrophic changes in the background gastric mucosa. Magnifying endoscopy with narrow‐band imaging (M‐NBI) revealed that the surrounding area had a regular pit pattern, while the redness area around the depression showed a heterogeneous arrangement of irregular villi and pit pattern without a clear demarcation line. Some of the structures were obscured in the depression (Figure 1b). Endoscopic ultrasonography (EUS) demonstrated a heterogeneous tumor with cystic areas in the third layer but preservation of the fourth layer (Figure 1c). An upper gastrointestinal X‐ray series showed a SMT in the upper gastric body with an irregular depression on its surface (Figure 1d). A biopsy specimen taken from the redness area around the depression showed a well to poorly differentiated adenocarcinoma. Since the adenocarcinoma had a SMT‐like morphology, preoperative differential diagnoses included gastric carcinoma with lymphoid stroma (GCLS), gastric adenocarcinoma of fundic gland type (GAFG), adenocarcinoma arising from heterotopic gastric mucosa, and adenocarcinoma arising from a gastric duplication cyst. GCLS and GAFG were ruled out based on the EUS findings of accompanying cystic components. As the cyst wall was not a multi‐layered structure, adenocarcinoma arising from a gastric duplication cyst was excluded as well. The ultimate preoperative diagnosis was adenocarcinoma arising from heterotopic gastric mucosa. Contrast computed tomography did not detect any metastasis. Due to the presence of a poorly differentiated component and the tumor's location in the submucosal layer on EUS, total laparoscopic gastrectomy with lymph node dissection was performed. The surgical specimen revealed an 18 × 13 mm SMT with a depression at the top (Figure 2). Histological examination of the SMT showed a submucosal mass of lobulated mucosa of gastric pyloric type, which had a centrally located lumen and was surrounded by smooth muscle communicating with the muscularis mucosa and confirmed the diagnosis of GHIP. Focal intramucosal adenocarcinoma of a well to poorly differentiated and signet ring cell type was found in the GHIP (Figure 3). On immunohistochemistry, the GHIP was positive for MUC5AC and MUC6, indicating differentiation of the pyloric gland mucosa. The cancerous area was also positive for MUC5AC, partly positive for MUC6, negative for MUC2, and displayed a gastric mucin phenotype and increased expression of Ki67 (Figure 4). No lymphovascular invasion or lymph node metastasis was found. These features suggested a diagnosis of adenocarcinoma arising in GHIP (U, Gre, pT1a(M), ly0, v0, pN0, M0, P0, CY0, H0, stageIA according to the Japanese classification of gastric carcinoma^3^). No recurrence has been reported during 4 years of postoperative follow‐up. It was noteworthy that during the 8 years of annual EGD for the gastric SMT, the size and height of the lesion had never changed remarkably (Figure 5).

## DISCUSSION

We encountered the rare case of a gastric adenocarcinoma arising from GHIP requiring total gastrectomy after a long and uneventful follow‐up period of 8 years.

GHIP is characterized by marked submucosal glandular proliferation associated with cystic dilatation that is accompanied by dendritic proliferation of the smooth muscle bundle.^1^ GHIP is rare, being found in less than 1% of all gastric polyps.^4^ Although diffuse heterotopic gastric glands in the submucosa are considered paracancerous lesions,^5^ the precise association of gastric carcinoma and GHIP remains controversial.

It was unclear whether the carcinoma in this case had existed from the first identification of the SMT or had become cancerous during follow‐up. Considering the absence of malignant findings in previous biopsies and a report on early‐stage gastric cancer progressing to advanced gastric cancer within 34 months,^6^ as well as the fact that poorly differentiated components were present, it was likely that the carcinoma had developed during the disease course. Although there are reports of cancerization with an increase in size of GHIP,^7^ no obvious morphological changes were observed in our patient during an 8‐year period. Hence, it may be difficult to diagnose carcinogenesis originating from GHIP by white‐light endoscopy only.

Since GHIP presents as an SMT‐like lesion covered with normal gastric mucosa, assessment by EUS is useful for detecting GHIP and visualizing hyperechoic lesions, including small cystic hypoechoic spots, in the third layer.^8^ However, EUS alone may be insufficient for distinguishing carcinoma from non‐neoplastic GHIP. In this case, M‐NBI was useful for diagnosis since it could detect an irregular microsurface pattern and enable a targeted biopsy‐diagnosed carcinoma. However, most tumors are not exposed on the surface, and it is considered that the mucosal changes are due to tumor infiltration near the surface layer rather than the tumor itself. It therefore remains difficult to diagnose carcinoma arising from GHIP using M‐NBI preoperatively depending on the location of the tumor. En bloc resection with endoscopic submucosal dissection (ESD) has also been performed for the accurate diagnosis and treatment of gastric cancer arising from GHIP as well as GHIP itself^9^, ^10^; however, the safety and therapeutic efficacy of ESD for a SMT predominantly located in the submucosa have not yet been established. Aggressive histological examination is important in GHIP, and both the diagnosis and treatment strategy should be carefully considered. In the present case, a biopsy specimen showed poorly differentiated tumor components and the tumor was located in the submucosal layer by EUS, which prompted us to select surgical resection. The evaluation of more cases is necessary to clarify the risk of carcinogenesis in GHIP and the diagnosis and treatment of GHIP‐associated gastric cancer.

## CONFLICT OF INTEREST

The authors declare no conflict of interest.

## ETHICS STATEMENT

All procedures performed in this case were in accordance with the ethical standards of the institutional committee and with the Helsinki declaration and its later amendments or comparable ethical standards. An informed consent was obtained from the patient for the publication of this case report.

## FUNDING INFORMATION

None.
